# Crepidtumines A and B, Two Novel Indolizidine Alkaloids from *Dendrobium crepidatum*

**DOI:** 10.1038/s41598-020-66552-2

**Published:** 2020-06-12

**Authors:** Xiaolin Xu, Xingyue Chen, Runmei Yang, Zesheng Li, Houguang Zhou, Yanbing Bai, Meng Yu, Biao Li, Gang Ding

**Affiliations:** 1 Institute of Medicinal Plant Development, Chinese Academy of Medical Sciences and Peking Union Medical College, Beijing, 100193 P. R. China; 2 Yunnan Dehong Institute of Tropical Agricultural Science, Dehong 678600, China, Dehong, 678600 People’s Republic of China

**Keywords:** Biochemistry, Carbohydrates

## Abstract

Two new indolizidine alkaloids crepidatumines A (**1**) and B (**2**) together with the stereoisomer of dendrocrepidine B (**3**) and known analog dendrocrepine (**4**) were isolated from *D. crepidatum*. Their structures were determined by HR-ESI-MS, NMR, and Electronic Circular Dichroism (ECD) experiments together with comparison of analogues. Compound (**1**) possess a (5/6/6/5) tetra-hetero-cyclic ring, whereas compound (**2**) contains a tricyclic system with an unusual bridged ring, which are the first report in Nature. The biological evaluation revealed that dendrocrepine (**4**) displayed a potent hypoglycemic effect *in vitro*.

## Introduction

**Figure 1:**
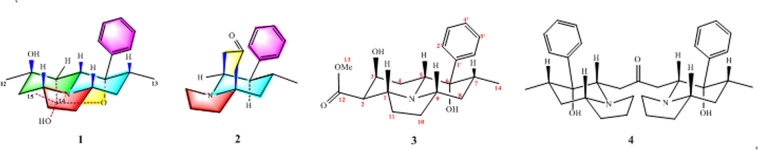
*Dendrobium crepidatum* and two new indolizidine alkaloids crepidatumines A (1) and B (2). Fig. 1. Structures of 1–4.

*Dendrobium* genus, a large member of Orchid plant with more than 1500 species, is distributed mainly in tropical and subtropical areas of Asia and Oceania^1^. Seventy-six species including two variants are found in the southern region of the Qin Ling mountain and Huai He river, China^2^. As one of the most famous Traditional Chinese Medicine (TCM), *Dendrobium* genus has a wide range of biological effects, for example, maintaining gastric tonicity, enhancing production of body fluid, relieving and curing symptoms of dryness, and of body heat^3^, and it also displays anti-tumor, antioxidation, immuno-enhancement and anti-inflammatory bioactivities^4–6^. In ancient time of China, this genus is named as one of “Nine Fairy Grasses”, which is very precious and rare. More than 300 compounds including polysaccharides, sesquiterpenoids, alkaloids, flavonoids, bibenzyls and phenanthrenes have been isolated from different *Dendrobium* species^7–10^. *D. crepidatum* is a unique species with very beautiful flowers growing in warm and humid environments at an altitude of 500~1500 meters, which has diverse functions including moistening the lung, eliminating asthenia heat, and diminishing inflammation^11,12^. So far, there were only several indolizine-type alkaloids and two stilbene derivatives isolated from this medicinal plant^13–15^. Indolizidine alkaloids can be divided into two major groups including phenanthroindolizidines and simple indolizidines. This member of alkaloids displays a large range of biological effect such as glycosidase inhibition, immune modulation, anti-viral and anti-cancer activities^16–18^. During our ongoing research to mine new/novel bioactive secondary metabolites from TCM plants, two indolizidine alkaloids crepidatumines A (**1**) and B (**2**) with new skeletons together with the stereoisomer of dendrocrepidine B (**3**) and dendrocrepine (**4**) were isolated from *D. crepidatum* (Fig. 1). Compound (**1**) possesses a novel (5/6/6/5) tetra-hetero-cyclic structure, whereas compound (**2**) contains a unique bridged-ring system. In this report, the isolation, structural elucidation, and biological activities of these compounds were present.

**Figure 2:**
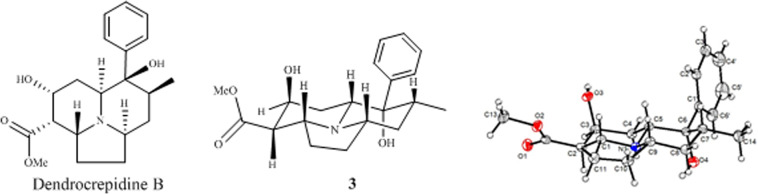
X-Ray crystal structure of 3.

The ^1^H, and ^13^C of compound **3** were similar with those of dendrocrepidine B, but the result from X-ray diffraction data determined its structure to be the stereoisomer of dendrocrepidine B, and this established its absolute configuration of **3** as 1*R*, 2*R*, 3*S*, 5*R*, 6*S*, 7*R*, and 9*S* (Fig. 2). The X-ray data of **3** was provided in Table 1.

**Table 1:** Crystal data and structure refinement for compound **3**.

Identification code	3
Empirical formula	C_20_H_27_NO_4_
Formula weight	345.42
Temperature / K	107.75(10)
Crystal system	orthorhombic
Space group	P2_1_2_1_2_1_
a / Å, b / Å, c / Å	6.6679(4), 10.7681(4), 24.2111(9)
α/°, β/°, γ/°	90, 90, 90
Volume / Å^3^	1738.38(13)
Z	4
ρ_calc_ / mg mm^−3^	1.320
μ / mm^−1^	0.737
F (000)	744
Crystal size / mm^3^	0.350 × 0.340 × 0.100
2Θ range for data collection	8.988 to 142.446°
Index ranges	−4 ≤ h ≤ 7, −11 ≤ k ≤ 13, −29 ≤ l ≤ 29
Reflections collected	5698
Independent reflections	3256[R(int) = 0.0271 (inf-0.9 Å)]
Data/restraints/parameters	3256/0/230
Goodness-of-fit on F^2^	1.054
Final R indexes [I > 2σ (I) i.e. F_o_ > 4σ (F_o_)]	R_1_ = 0.0397, wR_2_ = 0.1072
Final R indexes [all data]	R_1_ = 0.0421, wR_2_ = 0.1106
Largest diff. peak/hole / e Å^−3^	0.312/−0.240
Flack Parameters	−0.13(14)
Completeness	0.9984

## Results and Discussion

**Figure 3:**
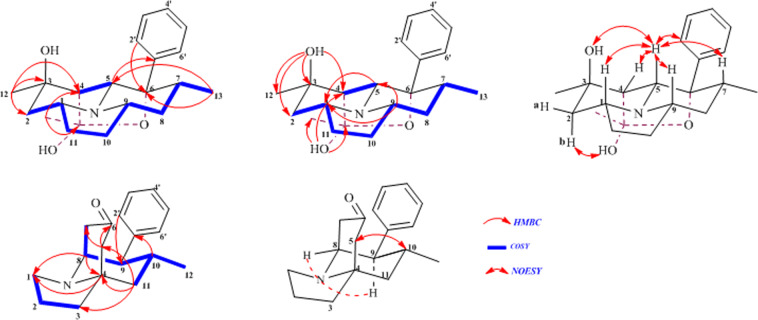
^1^H-^1^H COSY, Key HMBC and NOESY correlations of 1 and 2.

The molecular formula of crepidatumine A (**1**) was determined to be C_21_H_29_NO_3_ (8 degrees of unsaturation) on the basis of HRESI MS (*m/z* 344.2230 [M + H]^+^; Calcd 344.2226). The ^1^H, ^13^C, (Table 1) and HMQC NMR data of **1** (Table 2) revealed the presence of three methyls, four methylenes, five methines, three oxygenated carbons (one of which was suggested to be a hemiacetal carbon with chemical shift value *δ*_c_ = 104.6), and a mono-substituted phenyl ring. In addition, there are two singlet protons as free hydroxyl or amino groups. These data accounted for all the ^1^H and ^13^C-NMR resonance signals. Based on the degrees of unsaturation, it implied that **1** possessed a tetracyclic system. The ^1^H-^1^H COSY correlations established three isolated proton spin-systems including a mono-substituted phenyl unit, two fragments corresponding to –C-13-C-7-C-8-C-9-C-10-C-11-C-1-C-2–, and –C-4-C-5– (Fig. 3). The remaining connections were characterized by HMBC correlations. The correlations from 13-Me to C-6, from H-5 and H-7 to C-6 and C-1′, and from H-2′ (H-6′) to C-5, C-6 and C-7 confirmed that the C-6 was linked with C-5, C-7 and C-1′; correlations of H-5 with C-1 and C-9, and H-9 with C-1 established an indolizidine ring system; the HMBC cross peaks from 3-OH to C-2, C-3, C-4 and C-12 determined the connection of C-3 with C-2, C-4 and C-12, and a hydroxyl group was anchored at C-3; the correlations of 15-Me with C-4, C-14, and of 14-OH with C-4, C-14 and C-15 established the linkage of C-14 with C-4 and C-15, and of C-14 with an hydroxyl group. Considering the chemical shift value of C-14 (*δ*_c_ = 104.6), degrees of unsaturation and molecular formula, an ether bond must be shaped between C-6 and C-14 to form a unique hemiacetal group. Thus, the planar structure of **1** was characterized.

**Table 2:** NMR Spectroscopic Data of **1** and **2** in (DMSO-*d*_6_) (*δ* in ppm and J in Hz)^a,b^.

Pos	1		2	
1	56.8, CH	2.60, m	47.0, CH_2_	3.04, dt (8.4, 7.2) 2.97, dt (8.4, 8.4)
2	44.2, CH_2_	1.67, dd (13.2, 3.6) 1.46, t (13.2)	20.8, CH_2_	1.87, m
3	69.7, qC		38.8, CH_2_	1.63, m 1.66, m
4	56.0, CH	1.52, d (3.0)	62.1, qC	
5	67.8, CH	3.22, d (3.0)	47.9, CH_2_	2.35, br d (15.6) 2.20, br d (15.6)
6	84.2, qC		210.9, qC	
7	40.6, CH	1.85, m	36.2, CH_2_	2.33, dd (15.6, 6.0) 1.88, m
8	37.1, CH_2_	1.77, ddd (12.0, 4.8, 2.4) 1.17, dt (12.0, 12.0)	58.6, CH	3.33, overlapped
9	59.3, CH	2.45, m	52.3, CH	2.77, dd (4.2, 12.0)
10	29.1, CH_2_	1.85, m 1.36, m	27.0, CH	1.89, m
11	28.9, CH_2_	1.90, m 1.33, m	44.4, CH_2_	1.81, dd (4.8, 12.0) 1.26, t (12.0)
12	29.2, CH_3_	1.13, s	20.7, CH_3_	0.67, d (6.6)
13	15.5, CH_3_	0.61, d (6.6)		
14	104.6, qC			
15	27.8, CH_3_	1.45, s		
1'	145.8, qC		142.1, qC	
2′/6′	128.2, CH	7.34, br t (7.8)	129.0, CH	7.12, br d (7.8)
3′/5′	126.9, CH	7.43, m	128.9, CH	7.33, m
4′	126.6, CH	7.22, br t (7.8)	126.7, CH	7.22, m
3-OH		4.29, s		
14-OH		5.68, s		

^a^Assignments were based on HSQC, HMBC and ^1^H-^1^H COSY experiments.

^b^NMR spectroscopic data were recorded at 600 MHz (^1^H NMR), 150 MHz (^13^C NMR).

**Figure 4:**
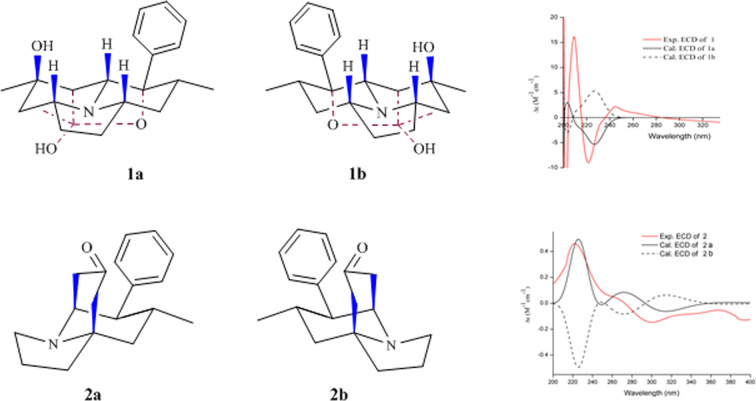
ECD of structure of 1 and 2.

The relative configuration of **1** was determined on the basis of analysis of NOESY correlations and coupling constants (Fig. 3). The correlations from H-5 with H-1, 3-OH, H-7, H-9, and H-2′ (H-6′) implied that H-1, 3-OH, H-5, H-7, and H-9 possessed pesudo-axial bonds, whereas the mono-substituted phenyl and 12-Me possessed pesudo-equatorial bonds; the coupling constant between H-4/H-5 was 3.0 Hz, which revealed that H-4 possessed pesudo-equatorial bond; H-2b was a broad triplet (t, *J* = 13.2 Hz) and H-2a was a doublet doublet (dd, *J* = 13.2, 3.6 Hz) confirmed that H-2b possessed pesudo-axial bond and H-2a possessed pesudo-equatorial bond; 15-Me had NOESY correlations with 12-Me, and H-2b had NOESY correlations with 14-OH implied that these groups were close each other in space (Fig. 3). Thus, the relative configuration of **1** was determined. Electronic circular dichroism (ECD) experiments combined with quantum-chemical calculations adopting time-dependent density functional theory (TDDFT) was used to establish the stereochemistry of crepidatumine A (**1**). The theoretical calculation of ECD was conducted in MeOH using Time-dependent Density functional theory (TD-DFT) at the B3LYP/6-311 + g (d, p) level for all conformers of compounds 1. To get the final spectra, the simulated spectra of the conformers were averaged according to the Boltzmann distribution theory and their relative Gibbs free energy (ΔG). By comparing the experiment spectrum with the calculated ECD spectra, the stereochemistry for **1** was determined to be 1*R*, 3*S*, 4*R*, 5*R*, 6*S*, 7*R*, 9*S*, 14*R* (Fig. 4) same as that of its analogue **3**, of which stereochemistry was determined by X-ray diffraction experiment.

The HRESIMS (*m/z* 270.1857 [M + H]^+^, Calcd 270.1858) gave the molecular formula of crepidatumine B (**2**) to be C_18_H_23_NO. Analysis of the NMR spectra revealed that compound **2** (Table 2) also possessed an indolizidine ring coupled a mono-substituted phenyl unit same as found in structures **1** and **3** (Fig. 3). The ^1^H-^1^H COSY spectrum linked C-7 and C-8; the HMBC correlations from 11-CH_2_- to C-4 and C-5, from 5-CH_2_- to C-6 and C-7 established the fragment of–C-4-C-5-C-6-C-7–, which formed a bridged-ring fused with a indolizine ring to shape the tetrahydro-1*H*-5,8a-ethanoindolizin-7(*8H*)-one carbon skeleton. Thus, the planar structure of **2** was characterized. The relative configuration of **2** was established based on the coupling constant analysis and NOESY correlations. The coupling constant of H-9 (*J* = 4.20, 12.0 Hz) together with the NOESY correlation from H-8 and H-9 confirmed that H-9, H-10 and –C-8-C-7– possessed pesudo-axial bonds, and H-8 possessed pesudo-equatorial bond; the NOESY correlation between H-10 with 5-CH_2_ revealed that –C-4-C-5– possessed pesudo-axial bond. The absolute configuration was determined to be 4 *S*, 8 *S*, 9 *S*, and 10 *R* on the basis of comparison of CD and ECD spectra of **2** (Fig. 4).

**Figure 5:**
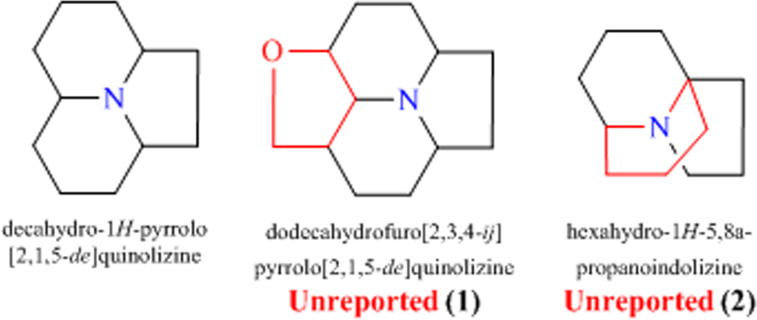
Key skeletons of 1 and 2.

Crepidatumine A (**1**) possesses an unusual dodecahydrofuro[2,3,4-ij] pyrrolo[2,1,5-de] quinolizine skeleton, which is formed by a decahydro-1*H*-pyrrolo[2,1,5-de] quinolizine ring coupled a tetrahydrofuran ring. More importantly, there are eight stereocenters (six carbons C-3-C-4(C-14)-C-5-C-6-C-7 are continued) and a hemiacetal hydroxyl group in the unique (5/6/6/5) tetra-hetero-cyclic system of crepidatumine A (**1**), which will be a potential star molecule to organic synthesis. Crepidatumine B (**2**) possess a unique tricyclic indolizine-type skeleton with an unusual bridged ring, which is the first report in Nature (Fig. 5).

**Figure 6:**
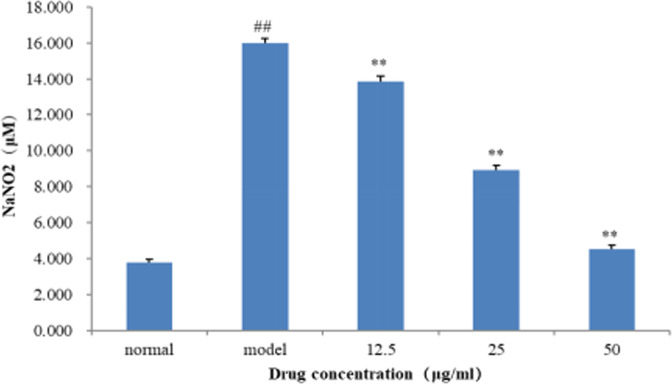
Effect of TAD on the viability of RAW 264.7 macrophages. Data are expressed as mean ± SD (n = 3). **p < 0.01 compared with the normal group; **p < 0.01 compared with the model (LPS-treated) group.

**Figure 7:**
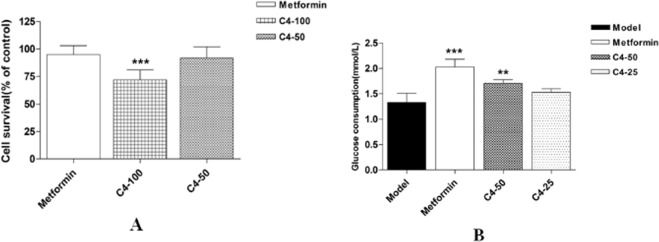
Effect of compound 4 (C4) on cell viability (**A**) and glucose consumption (**B**) in HepG2 cell. Data are shown as the mean ± SD (n = 6). **p < 0.01, ***p < 0.001 versus control (**A)** or model (**B**).

Compounds **1–4** were tested the anti-inflammatory activity in RAW 264.7 cells without biological effects, whereas the total alkaloids of *D. crepidatum* (TAD) inhibited the NO production induced by LPS in RAW 264.7 cells in a dose-dependent fashion without cytotoxicity (Fig. 6)^19^. Thus, it is merited to go to mine bioactive natural products from these total alkaloids. In addition, compounds **1–4** were evaluated the cytotoxic activities against three cancer cell lines A459, and HCT116, and gram-positive bacteria Bacillus subtilis 63501, *Staphylococcus aureus* 29213, and gram-negative bacterium *Escherichia coli* 25922 without bioactivities. The high glucose model of HepG2 cells was used to evaluate the hypoglycemic effect of compound **4**. As a result, compared with the model group, this compound significantly increased the glucose consumption by 29% at the concentrations of 50 μmol/L without cytotoxicity, which implying that this compound might be a potent molecule as a hypoglycemic agent (Fig. 7).

## Conclusion

Four indolizidine alkaloids including two new compounds crepidatumines A (**1**) and B (**2**) together with the stereoisomer (**3**) of dendrocrepidine B and known analog dendrocrepine (**4**) were isolated from *D. crepidatum* Compound (**1**) possess a unusual (5/6/6/5) tetra-hetero-cyclic ring, whereas compound (**2**) contains a unique tricylic system with an unusual bridged ring, which are the first report in Nature. Compounds **1–4** did not display anti-inflammatory, anti-microbial and cytotoxic activities, but compound **4** could increase glucose consumption, implying that **4** might be a potent molecule as a hypoglycemic agent.

## Methods

### General experimental procedures

Optical rotations were measured on a PerkinElmer 241 polarimeter, and UV data were determined on a ThermoGenesys-10S UV-vis spectrometer^19^. IR data were recorded using a Nicolet IS5FT-IR spectrophotometer. CD spectra were obtained on a JASCO J-810 spectrometer. ^1^H and ^13^C NMR data were acquired with a Bruker 600 spectrometer using solvent signals (DMSO-*d*_6_; *δ*_H_ 2.50/*δc* 39.5) as references. the HMQC and HMBC experiments were optimized for 145.0 and 8.0 Hz, respectively. HRESIMS were obtained using a TOF-ESI-MS (Waters Synapt G2, USA). Semipreparative HPLC separation was carried out using a Lumtech instrument packed with a YMC-Pack ODS-A column (5 μm, 250 × 10 mm). Sephadex LH-20 (Pharmacia Biotech AB, Uppsala, Sweden) and silica gel (200–300 mesh) (Qingdao Marine Chemical Plant, Qingdao, China) were used.

### Plant material

The stems of *D. crepidatum* were collected from Ruili Resource Nursery of Dendrobium Germ Plasm and Resources, the Ministry of Agriculture and Rural Affairs of the People’s Republic of China in August 2017^19^. The sample was identified by one of the co-authors Ze-Sheng Li from Yunnan Dehong Institute of Tropical Agricultural Science. A voucher specimen was deposited in the herbarium of the Institute of Medicinal Plant Development, Chinese Academy of Medical Sciences.

### Extraction and isolation

The dried stems of *D. crepidatum* (9.0 kg) were extracted under reflux with 95% ethanol (50 L × 3 h, three times)^19^. The combined extract was suspended with water, and extracted with petroleum ether and CH_2_Cl_2_ three times separately. The fraction of CH_2_Cl_2_ was concentrated into extractum, and dissolved in 5% hydrochloric acid filtered, then adjusted to pH 10 with ammonia water. Finally, it was extracted by CH_2_Cl_2_ three times at room temperature. The CH_2_Cl_2_ extract was obtained the total alkaloids 90.0 g of crude extract. The original extract was fractionated on a silica gel CC eluted with petroleum ether- acetone (50:1, 40:1, 30:1, 20:1, 15:1, 10:1, 5:1, 2:1 and 0:1, v/v, each 6.6 L) to give five fractions (Fr.1 to Fr.5). Fr.2 (10.0 g) was fractionated on a silica gel column chromatography (CC) using petroleum ether-acetone isocratic elution (30:1) to afford six fractions (Fr.2.1-Fr.2.6) and the crystal compound 4 (4.3 g). Separation of Fr.2.4 (2.0 g) was separated over Sephadex LH-20 (CH_2_Cl_2_: MeOH/1:1) to give four fractions (Fr.2.4.1-Fr.2.4.4). Fr.2.4.2 (500.0 mg) was further purified by semi-preparative HPLC (60–100% MeOH-H_2_O for 30 min, v/v, 2 mL/min) to yield 1 (5.9 mg, *t*_R_ 29.8 min). Fr.2.4.3 (1.0 g) was purified by semi-preparative HPLC (60–100% MeOH-H_2_O for 30 min, v/v, 2 mL/min) to yield 2 (15.0 mg, *t*_R_ 26.0 min). Fr.2.5 (2.0 g) was further purified by semi-preparative HPLC (60–100% MeOH-H_2_O for 30.0 min, v/v, 2 mL/min) to yield 3 (4.0 mg, *t*_R_ 33.0 min).

Crepidatumine A (**1**): white powder; [α]_D_^25^ + 4.44 (*c* 0.1, MeOH); UV (MeOH) *λ*_max_ (log *ε*) 205 (3.70); IR (neat) *ν*_max_ 3433, 2965, 1209, 1032, 935, 755 cm^−1^; for ^1^H NMR and ^13^C NMR data see Table 1; Positive HR-ESI-MS: *m/z* 344.2230 (calcd. for C_21_H_30_NO_3_ [M + H]^+^, 344.2226).

Crepidatumine B (**2**): orange oil; [α]_D_^25^-26.0 (*c* 0.1, MeOH); UV (MeOH) *λ*_max_ (log *ε*) 205 (4.03); IR (neat) *ν*_max_ 2924, 2874, 1708, 1056, 1031, 704 cm^−^1; for ^1^H NMR and ^13^C NMR data see Table 1; Positive HR-ESI-MS: *m/z* 270.1857 (calcd. for C_18_H_24_NO [M + H]^+^, 270.1858).

### X-ray crystallographic analysis of 3

Upon crystallization from *n*-Hexane–CH_2_Cl_2_ (10:1) using the vapor diffusion method, colorless crystals were obtained for **3** C_20_H_27_NO_4_, M = 345.42, orthorhombic, a = 6.6679(4) Å, b = 10.7681(4) Å, c = 24.2111(9) Å, U = 1738.38(13) Å3, *T* = 107.75(10), space group P2_1_2_1_2_1_ (no. 19), Z = 4, *μ* (Cu Kα) = 0.737, 5698 reflections measured, 3256 unique (Rint = 0.0271) which were used in all calculations. The final wR (F2) was 0.1106 (all data).Crystallographic data for the structure of **3** has been deposited in the Cambridge Crystallographic Data Centre [**deposition number: CCDC 1908235**].

### Cell culture and sample treatment

The murine macrophage RAW 264.7 cell line was obtained from American Type Culture Collection (ATCC, Rockville, MD, USA)^20^. The cells were cultured at 37 °C in DMEM (Invitrogen, California, USA) supplemented with 10% heat-inactivated FBS (Hyclone, Utah, Logan, USA), 2 m ML - glutamine, penicillin G (100 IU/mL) and streptomycin (100 mg/mL) in a humidified atmosphere containing 5% CO_2_ and 95% air. The cells were incubated with total alkaloids of *D. crepidatum* (TAD) at different concentrations and then stimulated with LPS (10 ng/mL) for the indicated time. The stock solutions of TAD were prepared in dimethyl sulfoxide (DMSO), and the final concentration of DMSO was less than 0.5%.

The level of NO production was monitored by measuring the nitrite level in the culture medium. This was performed by mixing the medium with Griess reagent (1% sulfanilamide in 5% phosphoric acid and 0.1% *N*-1-naphthylethylenediamine dihydrochloride in water). The absorbance was measured at a wavelength of 540 nm after incubation for 10 min. The nitrite concentration was calculated with reference to a standard curve of sodium nitrite generated from known concentrations. Cell viability was assessed using an MTT assay.

### *In Vitro* Evaluation of Compound 4

Cell culture: Human hepatoma cells HepG2 were cultured in Dulbecco’s modified Eagle’s medium (DMEM, HyClone)^19^. DMEM was supplemented with 10% fetal bovine serum (Gibco) and 1% penicillin/streptomycin (HyClone) in a humidified atmosphere of 5% CO_2_ and 37 °C.

Assay for cell viability: Before the experiment, the assay for cell viability was determined with the cell counting kit-8 (CCK-8). HepG2 cells were seeded in 96-well plates as 2.5 × 10^3^ cells each well. Afterculturing for 24 h,the control group was added with serum-free medium, while the experimental groups were with the medium containingdifferent concentrations (50and 100 μmol/L) of compound **4** or 200 μmol/Lof metformin for another 24 h. Then the cells were treated with CCK-8 for 3 h. Finally, the absorbance was measured at 450 nm. The cell viability was calculated as the absorbance of each treated well divided by the control.

Assay for hypoglycemic activity: For the experiment, the cells were seeded in 96-well plates as 1 × 10^4^ cells each well. After culturing for 24 h, the medium containing different concentrations (25 and 50 μmol/L) of compound **4** were added for 24 h. The cells with 200 μmol/L metformin treatment were taken as positive control and the cells with phenol red-free DMEM as control. After the drug treatment, the glucose concentrations of the medium were determined with the glucose oxidase method. The glucose consumption of each well was obtained by subtracting the glucose concentrations of the experimental medium from the control group.

### ECD calculations of 1 and 2

Monte Carlo conformational searches were carried out by means of the Spartan’s 10 software using Merck Molecular Force Field (MMFF)^19^. The conformers with Boltzmann-population of over 5% were chosen for ECD calculations, and then the conformers were initially optimized at B3LYP/6-31 + g (d, p) level in MeOH using the CPCM polarizable conductor calculation model. The theoretical calculation of ECD was conducted in MeOH using Time-dependent Density functional theory (TD-DFT) at the B3LYP/6-311 + g (d, p) level for all conformers of compounds **1** and **2**. Rotatory strengths for a total of 50 excited states were calculated. ECD spectra were generated using the program SpecD is 1.6 (University of Würzburg, Würzburg, Germany) and GraphPad Prism 5 (University of California San Diego, USA) from dipole-length rotational strengths by applying Gaussian band shapes with sigma = 0.3 eV.

## Supplementary information

Supplementary Information.
