# Supplementary material for: Crepidtumines A and B, Two Novel Indolizidine Alkaloids from Dendrobium crepidatum
Source: Sci Rep. 2020 Jun 12;10:9564. doi: 10.1038/s41598-020-66552-2 (PMC7293321; doi:10.1038/s41598-020-66552-2)
Supplement: Supplementary file 1 — Supplementary Information. [file 41598_2020_66552_MOESM1_ESM.pdf]

# Crepidatumines A and B, Two Novel Indolizidine Alkaloids from *Dendrobium crepidatum*

Xiaolin Xu[a], Xingyue Chen[a], Runmei Yang[a], Zesheng Li[b], Houguang Zhou[b], Yanbing Bai[b], Meng Yu[a], Biao Li[a, *] and Gang Ding[a, *]

[a]*Institute of Medicinal Plant Development, Chinese Academy of Medical Sciences and Peking Union Medical College, Beijing, 100193, P. R. China*

[b]*Yunnan Dehong Institute of Tropical Agricultural Science, Dehong, 678600, P. R. China*

## Supporting Information

**Table of Contents**


**Fig S1.** IR spectrum of crepidatumine A (**1**)

**Fig S2.** UV spectrum of crepidatumine A (**1**) in $CH_3OH$

**Fig S3.** HRESIMS spectrum of crepidatumine A (**1**)

**Fig S4.** $^1$HNMR spectrum of crepidatumine A (**1**) in DMSO-$d_6$

**Fig S5.** $^{13}$C NMR spectrum of crepidatumine A (**1**) in DMSO-$d_6$

**Fig S6.** HSQC spectrum of crepidatumine A (**1**) in DMSO-$d_6$

**Fig S7.** $^1$H-$^1$H COSY spectrum of crepidatumine A (**1**) in DMSO-$d_6$

**Fig S8.** HMBC spectrum of crepidatumine A (**1**) in DMSO-$d_6$

**Fig S9.** NOESY spectrum of crepidatumine A (**1**) in DMSO-$d_6$

**Fig S10.** CD spectrum of crepidatumine A (**1**) in $CH_3OH$

**Fig S11.** IR spectrum of crepidatumine B (**2**)

**Fig S12.** UV spectrum of crepidatumine B (**2**) in $CH_3OH$

**Fig S13.** HRESIMS spectrum of crepidatumine B (**2**)

**Fig S14.** $^1$HNMR spectrum of crepidatumine B (**2**) in DMSO-$d_6$

**Fig S15.** $^{13}$C NMR spectrum of crepidatumine B (**2**) in DMSO-$d_6$

**Fig S16.** HSQC spectrum of crepidatumine B (**2**) in DMSO-$d_6$

**Fig S17.** $^1$H-$^1$H COSY spectrum of crepidatumine B (**2**) in DMSO-$d_6$

**Fig S18.** HMBC spectrum of crepidatumine B (**2**) in DMSO-$d_6$

**Fig S19.** NOESY spectrum of crepidatumine B (**2**) in DMSO-$d_6$

**Fig S20.** CD spectrum of crepidatumine B (**2**) in $CH_3OH$

**Fig S21.** $^1$HNMR spectrum of (**3**) in DMSO-$d_6$

**Fig S22.** $^{13}$C NMR spectrum of (**3**) in DMSO-$d_6$

**Table S1** NMR Spectroscopic Data of **3** in (DMSO-$d_6$) ($\delta$ in ppm and $J$ in Hz)

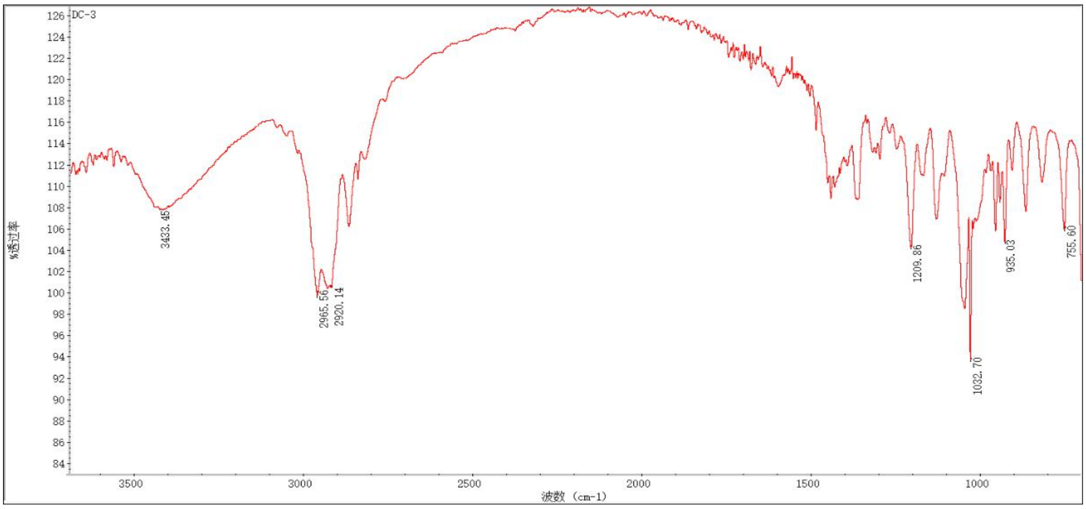


**Fig S1.** IR spectrum of crepidatumine A (**1**)

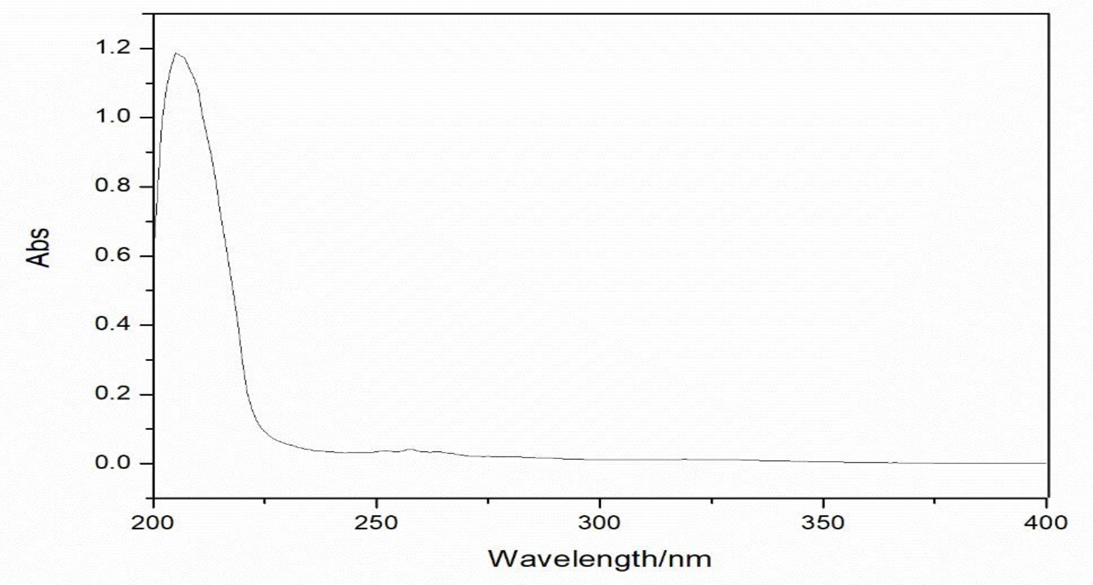


**Fig S2.** UV spectrum of crepidatumine A (**1**) in $CH_3OH$

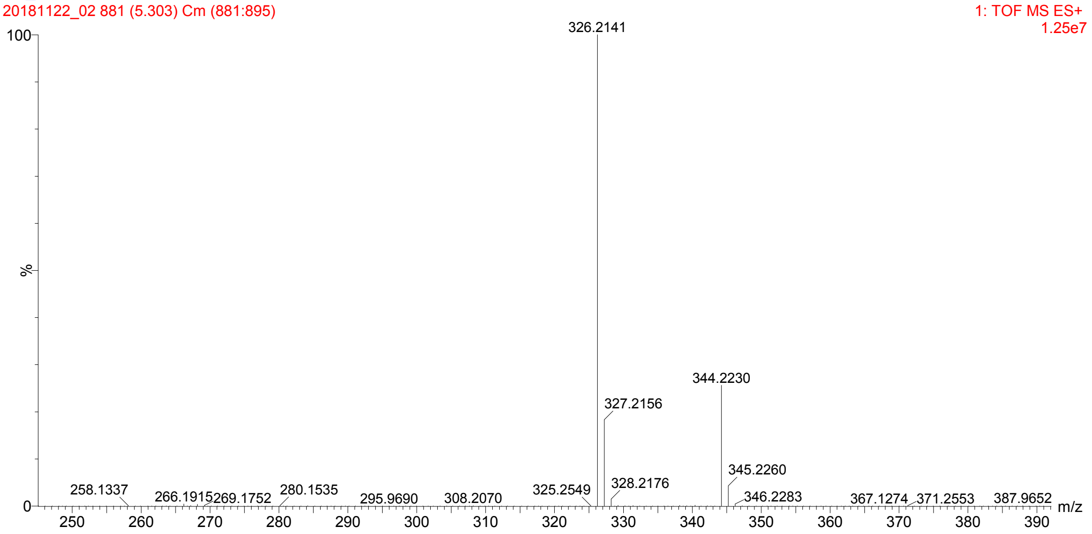


**Fig S3.** HRESIMS spectrum of crepidatumine A (**1**)

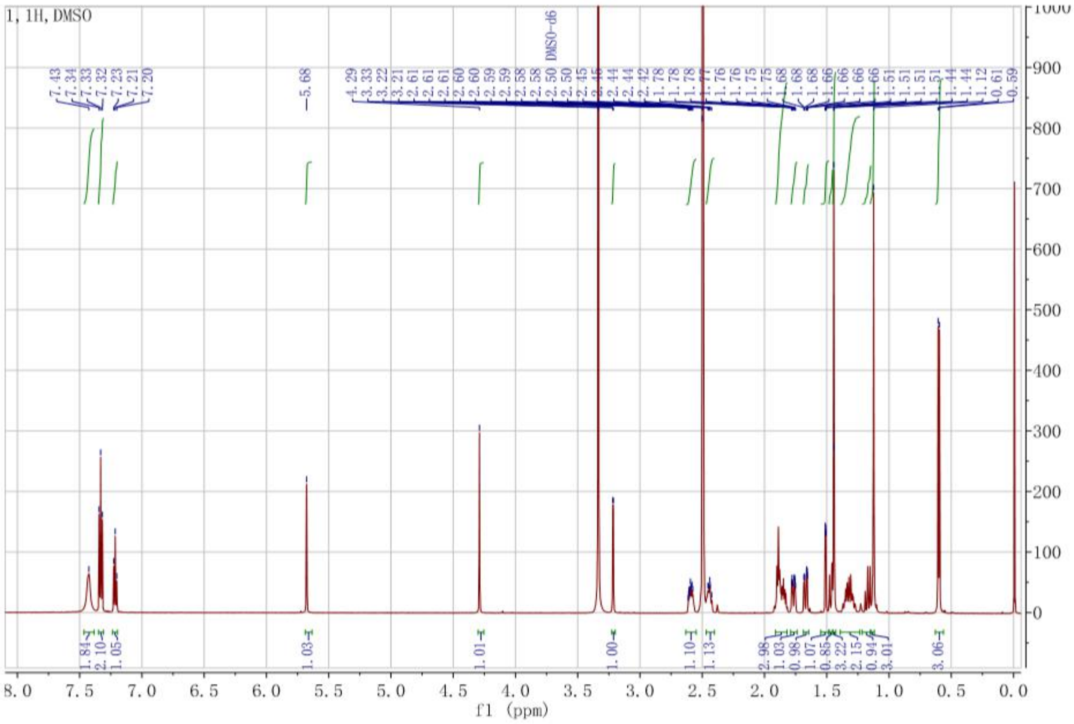


**Fig S4.** $^{1}$H NMR spectrum of crepidatumine A (**1**) in **DMSO-$d_6$**

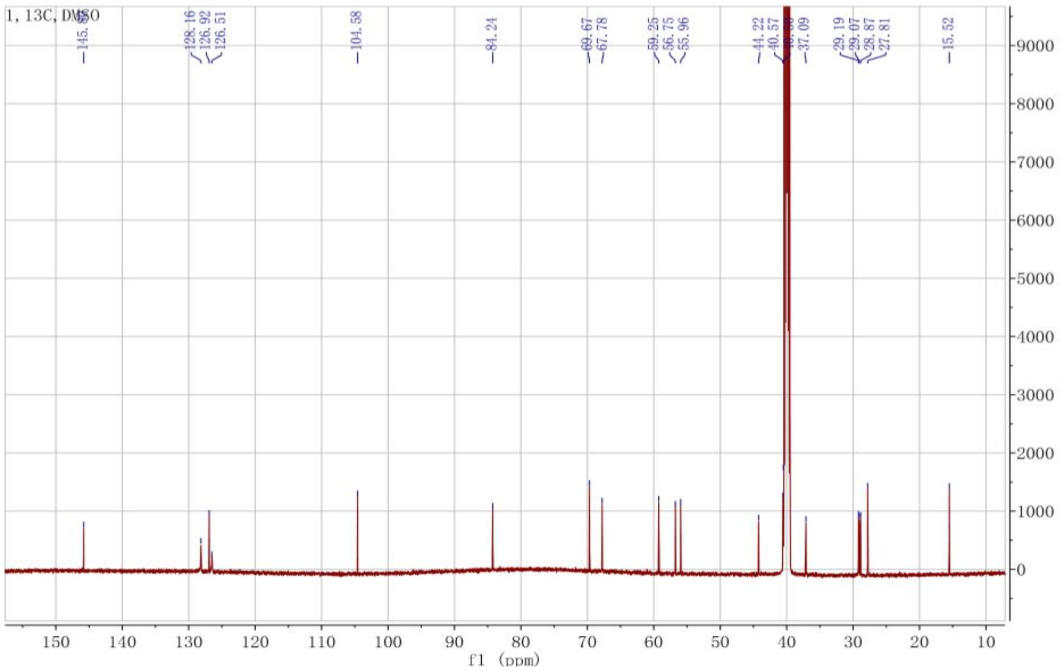


**Fig S5.** $^{13}$C NMR spectrum of crepidatumine A (**1**) in **DMSO-*d*$_6$**

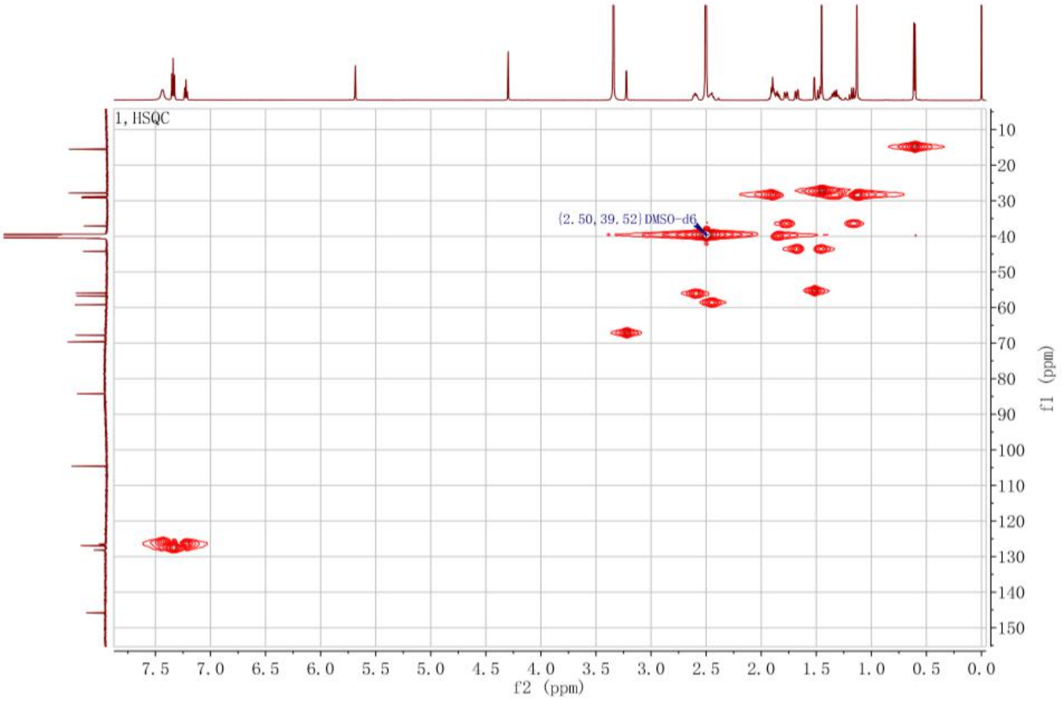


**Fig S6.** HSQC spectrum of crepidatumine A (**1**) in **DMSO-*d*$_6$**

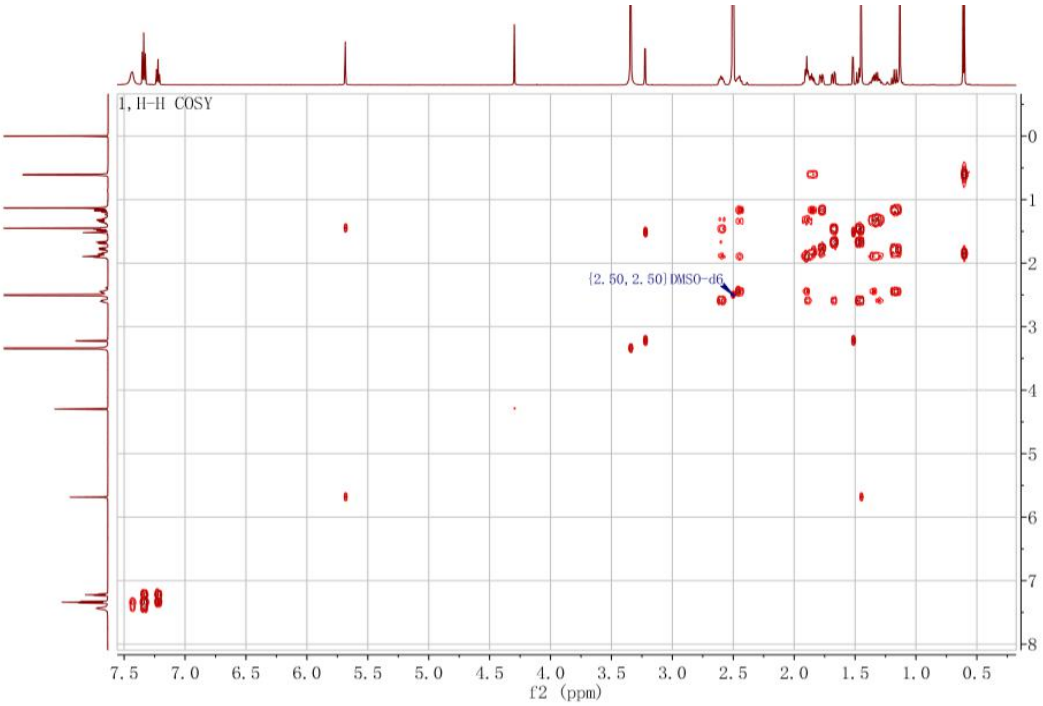


**Fig S7.** $^{1}H$-$^{1}H$ COSY spectrum of crepidatumine A (**1**) in **DMSO-*d*$_6$**

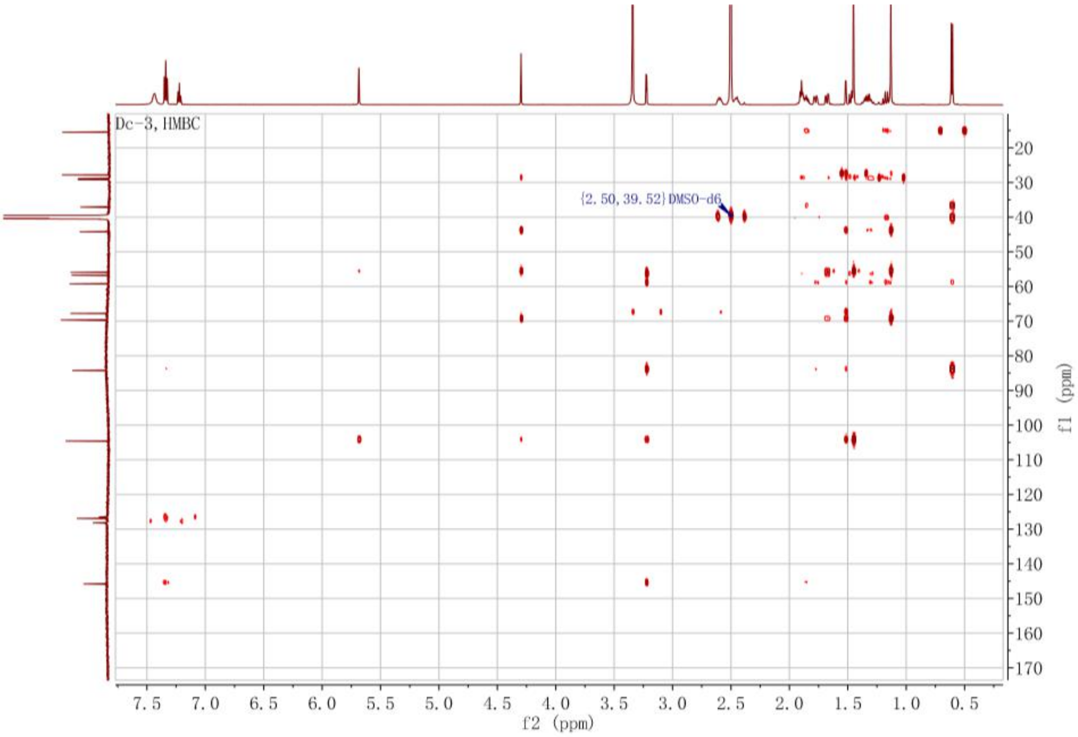


**Fig S8.** HMBC NMR spectrum of crepidatumine A (**1**) in **DMSO-*d*$_6$**

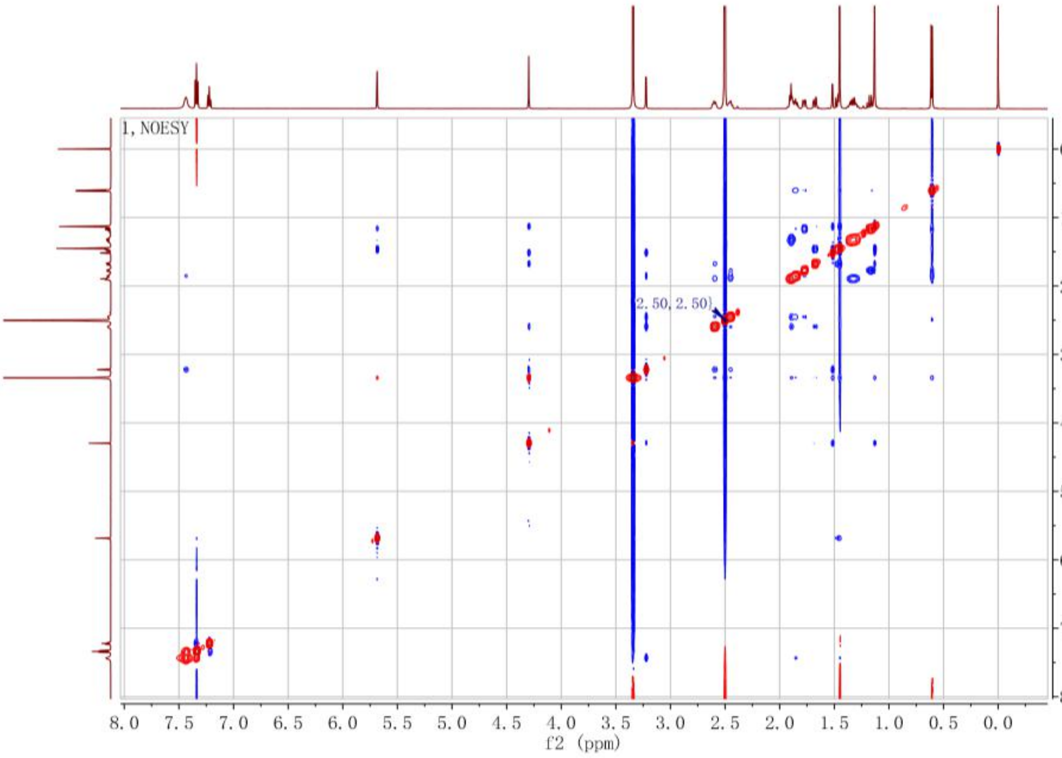


**Fig S9.** NOESY spectrum of crepidatumine A (**1**) in **DMSO-$d_6$**

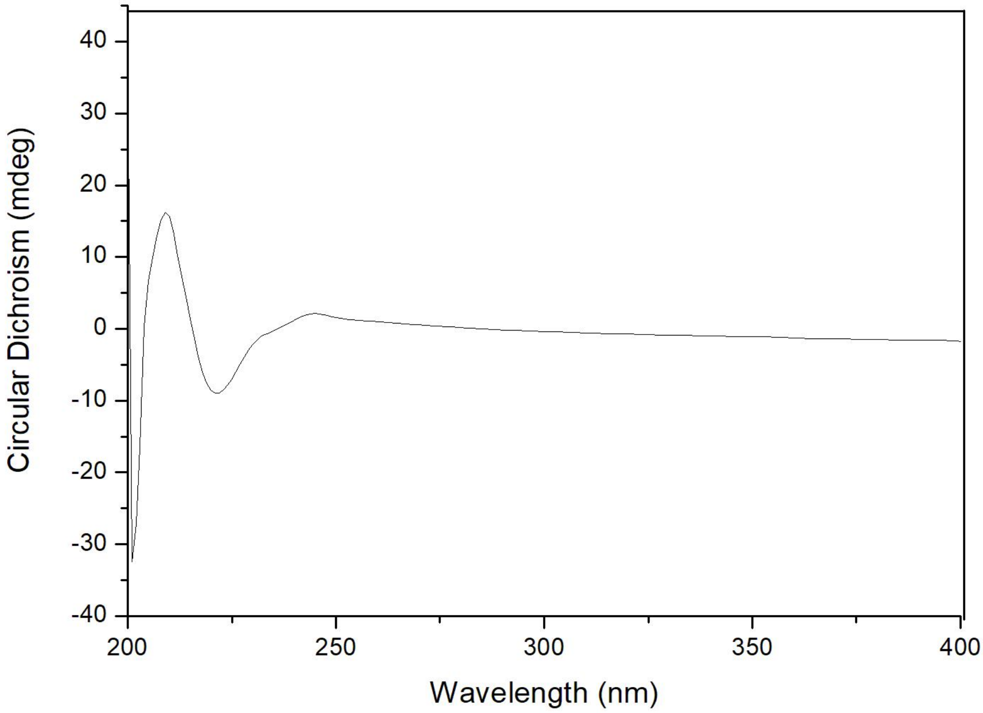


**Fig S10.** CD spectrum of crepidatumine A (**1**) in $CH_3OH$

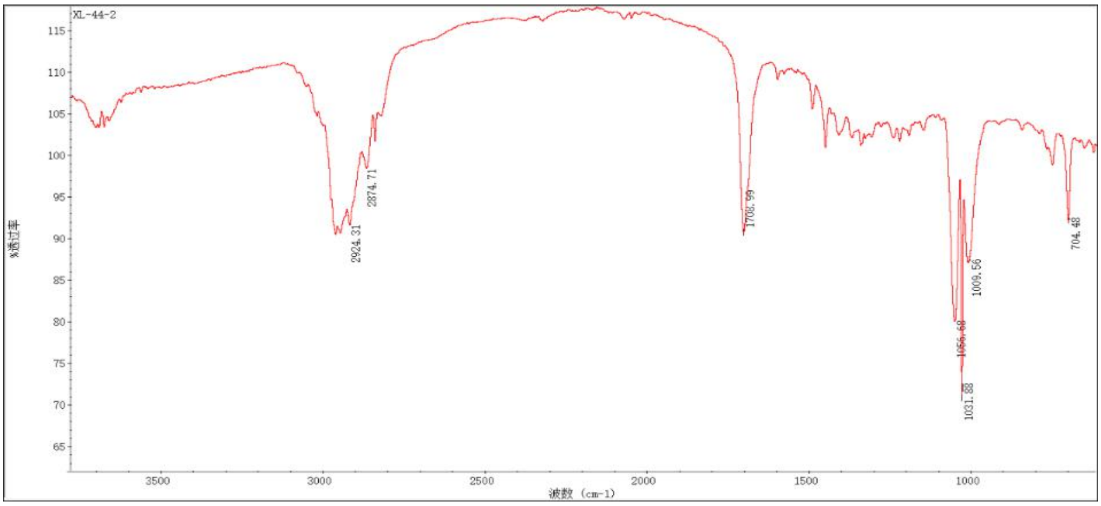


**Fig S11.** IR spectrum of crepidatumine B (**2**)

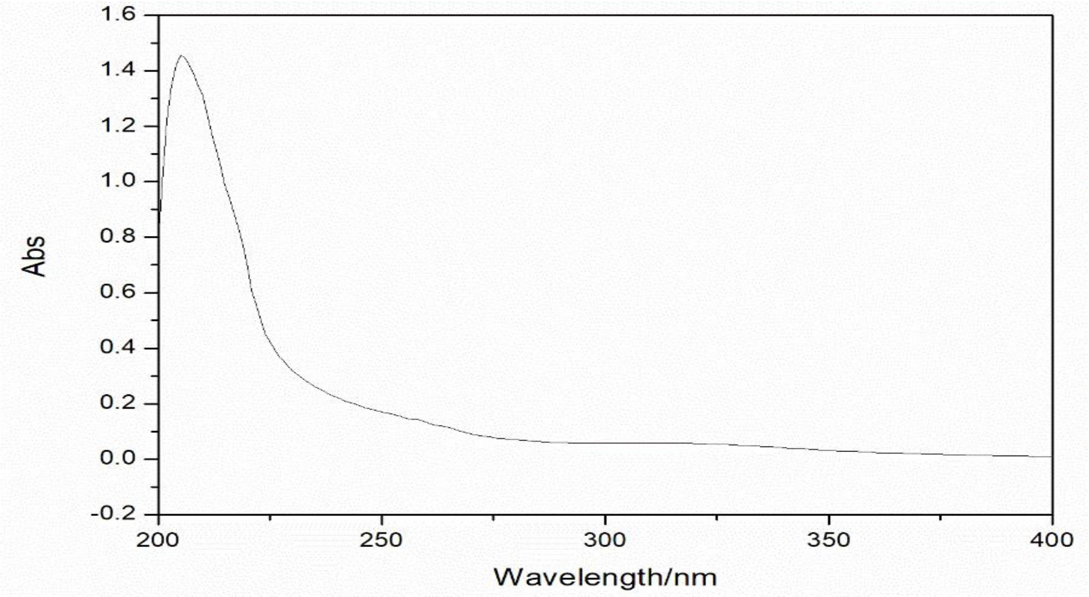


**Fig S12.** UV spectrum of crepidatumine B (**2**) in $CH_3OH$

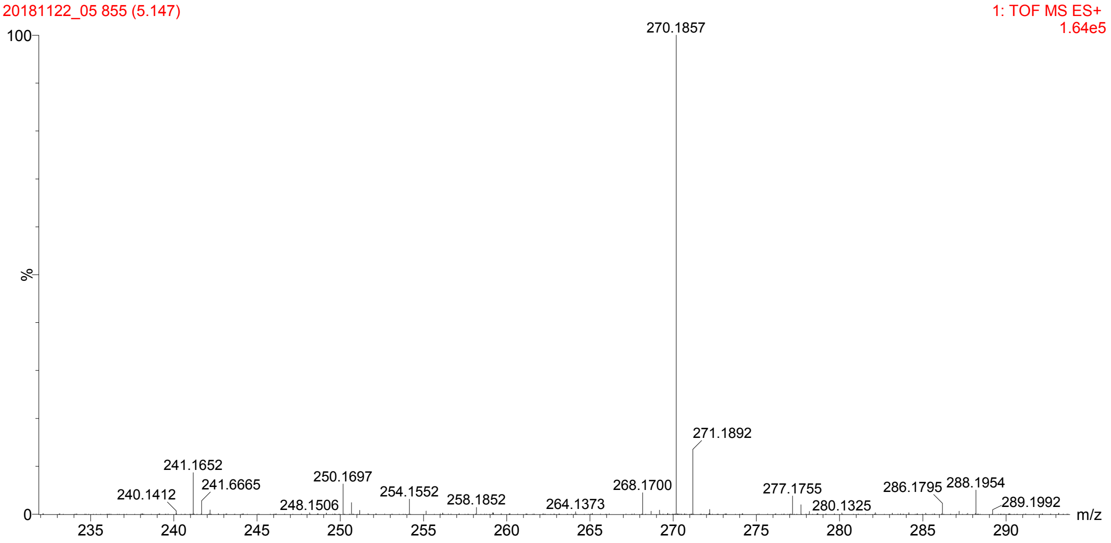


**Fig S13.** HRESIMS spectrum of crepidatumine B (**2**)

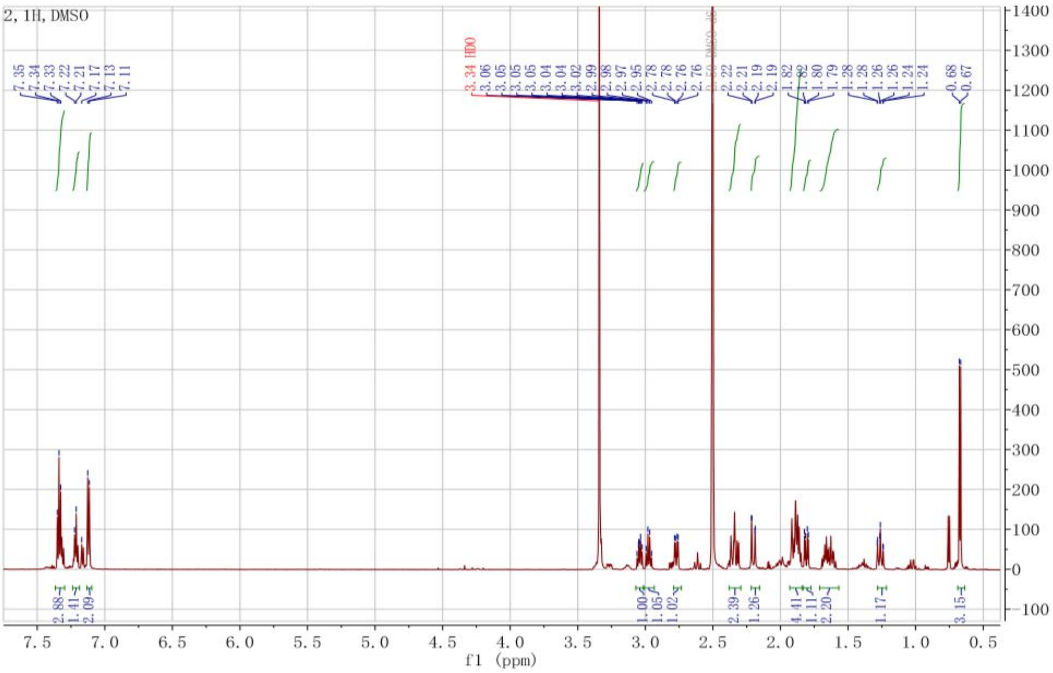


**Fig S14**. $^{1}$H NMR spectrum of crepidatumine B (**2**) in **DMSO-$d_6$**

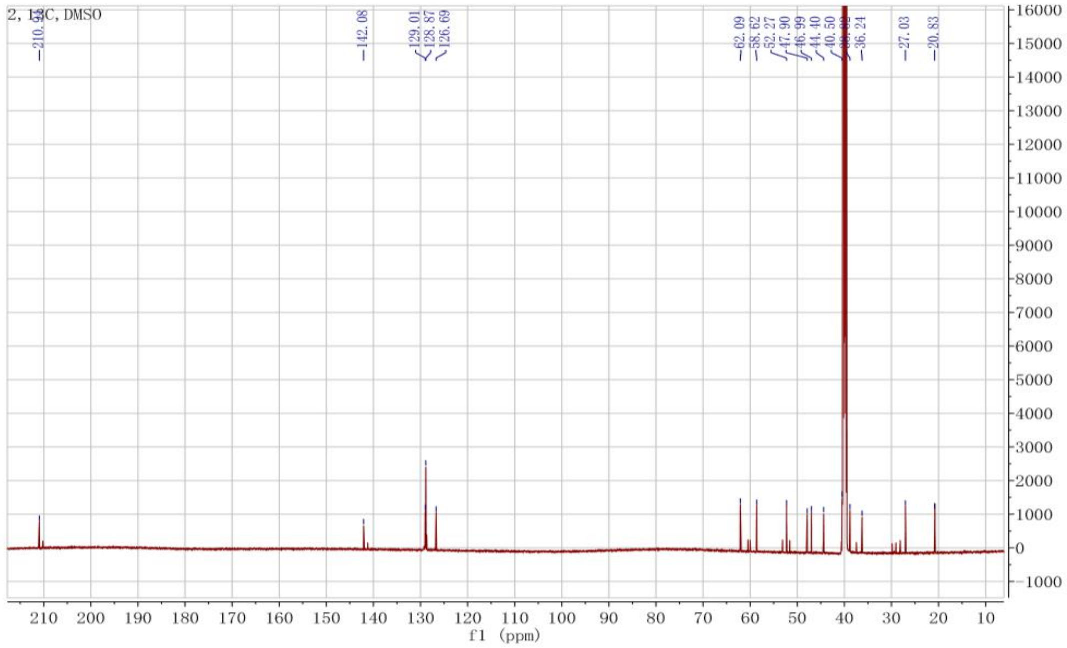


**Fig S15.** $^{13}C$ NMR spectrum of crepidatumine B (**2**) in **DMSO-*d*$_6$**

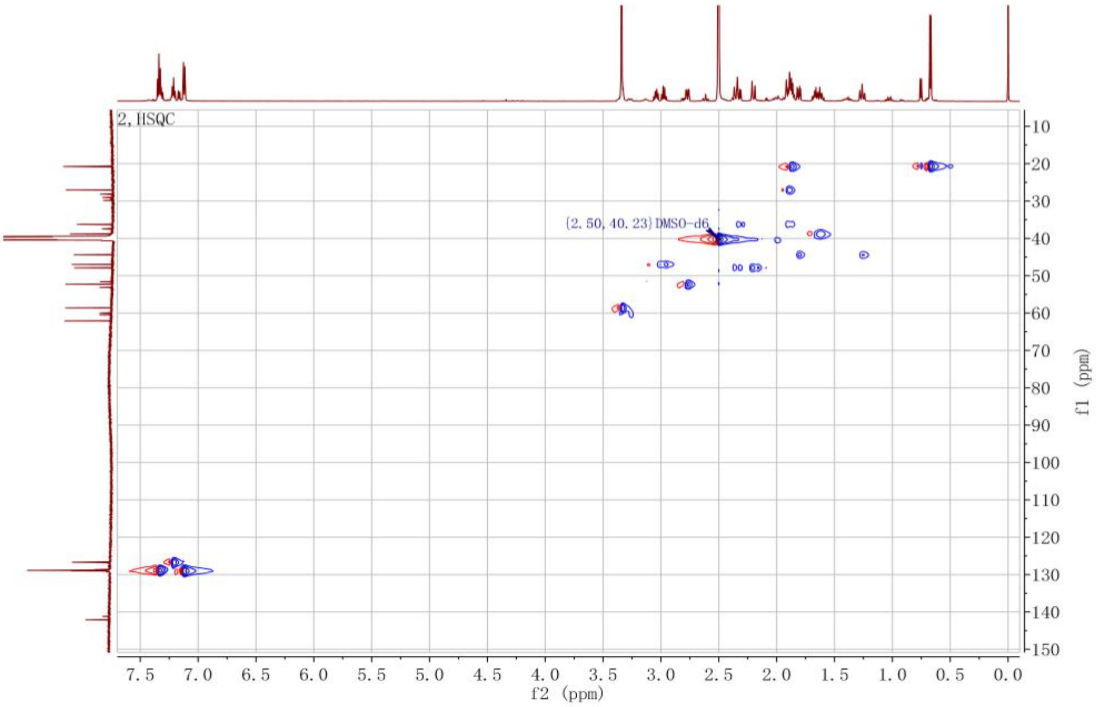


**Fig S16.** HSQC spectrum of crepidatumine B (**2**) in **DMSO-*d*$_6$**

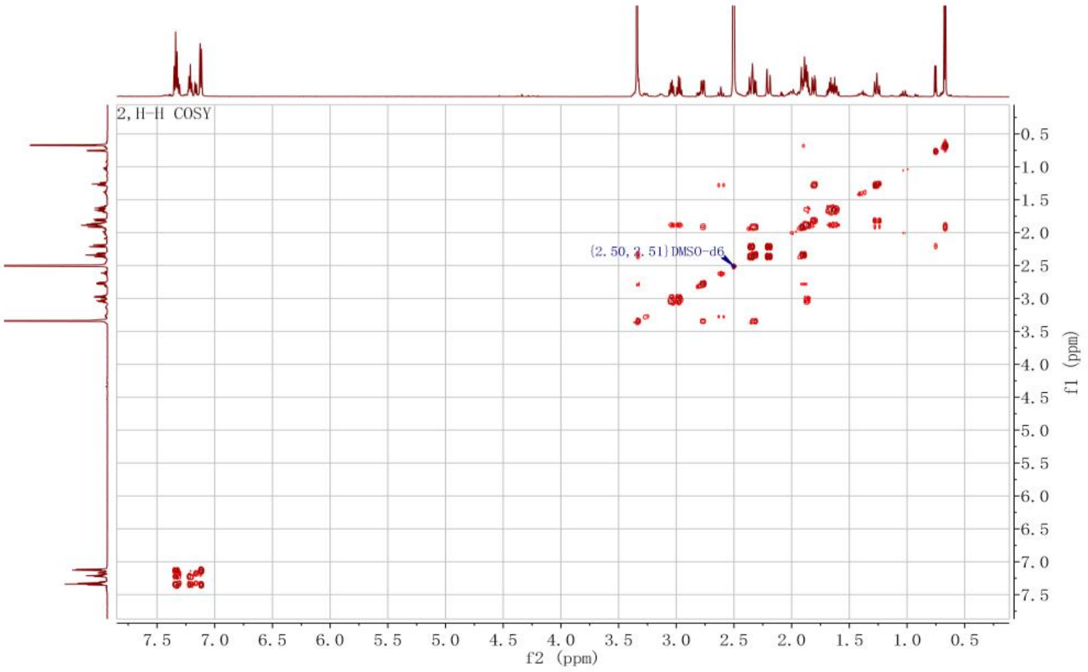


**Fig S17.** $^{1}H$-$^{1}H$ COSY spectrum of crepidatumine B (**2**) in **DMSO-*d*$_6$**

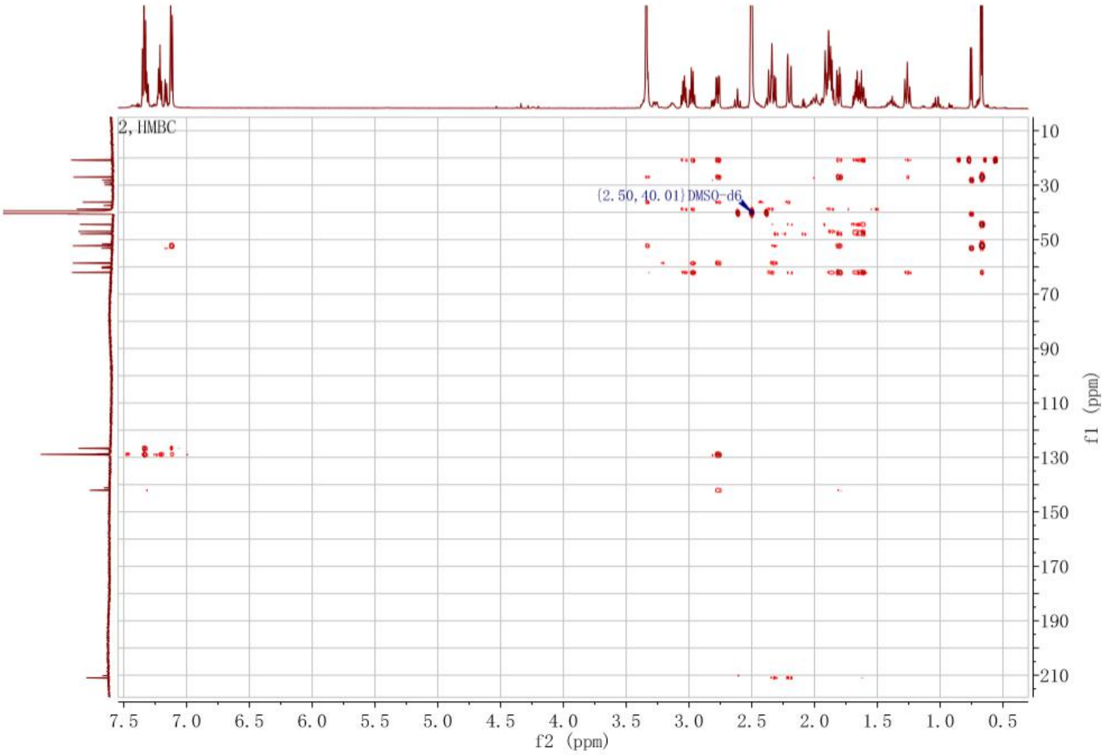


**Fig S18.** HMBC spectrum of crepidatumine B (**2**) in **DMSO-*d*$_6$**

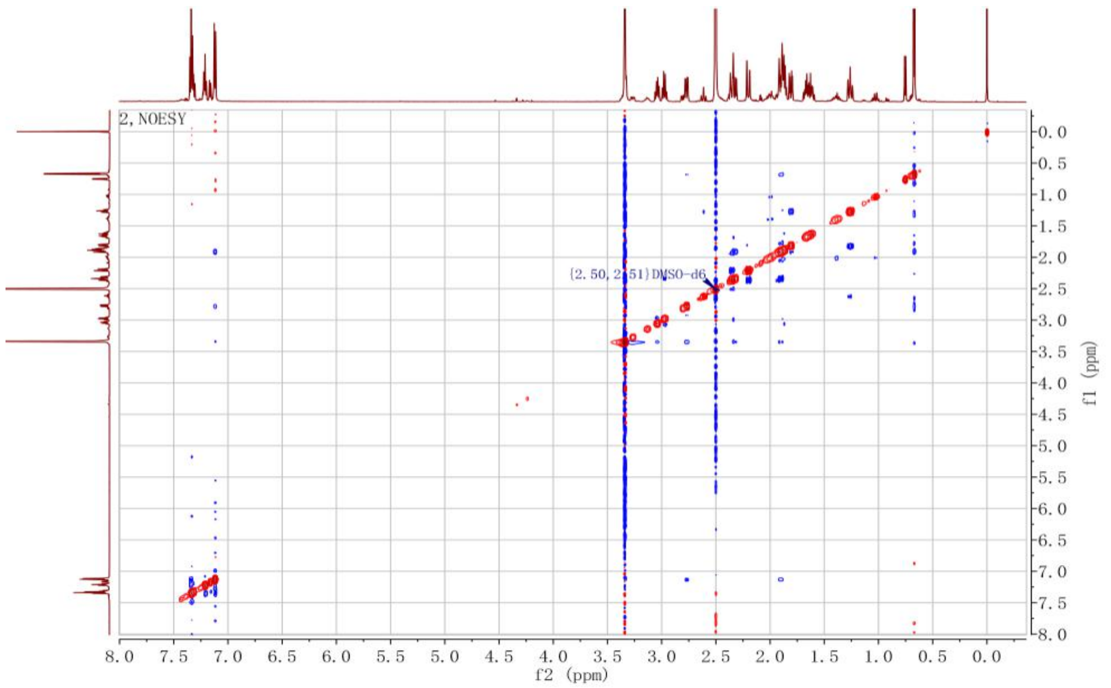


**Fig S19.** NOESY spectrum of crepidatumine B (**2**) in **DMSO-*d*$_6$**

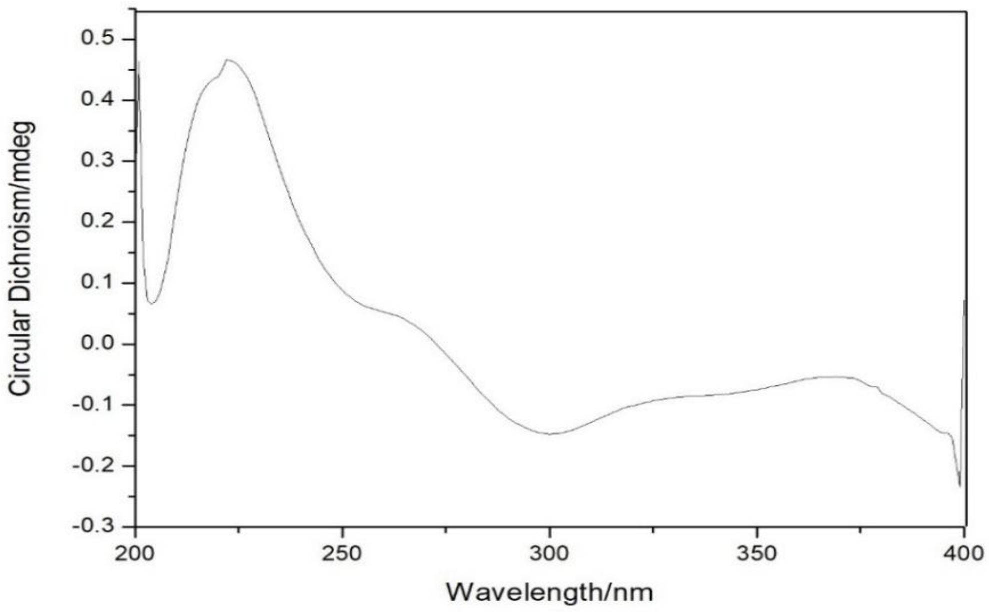


**Fig S20.** CD spectrum of crepidatumine B (**2**) in $CH_3OH$

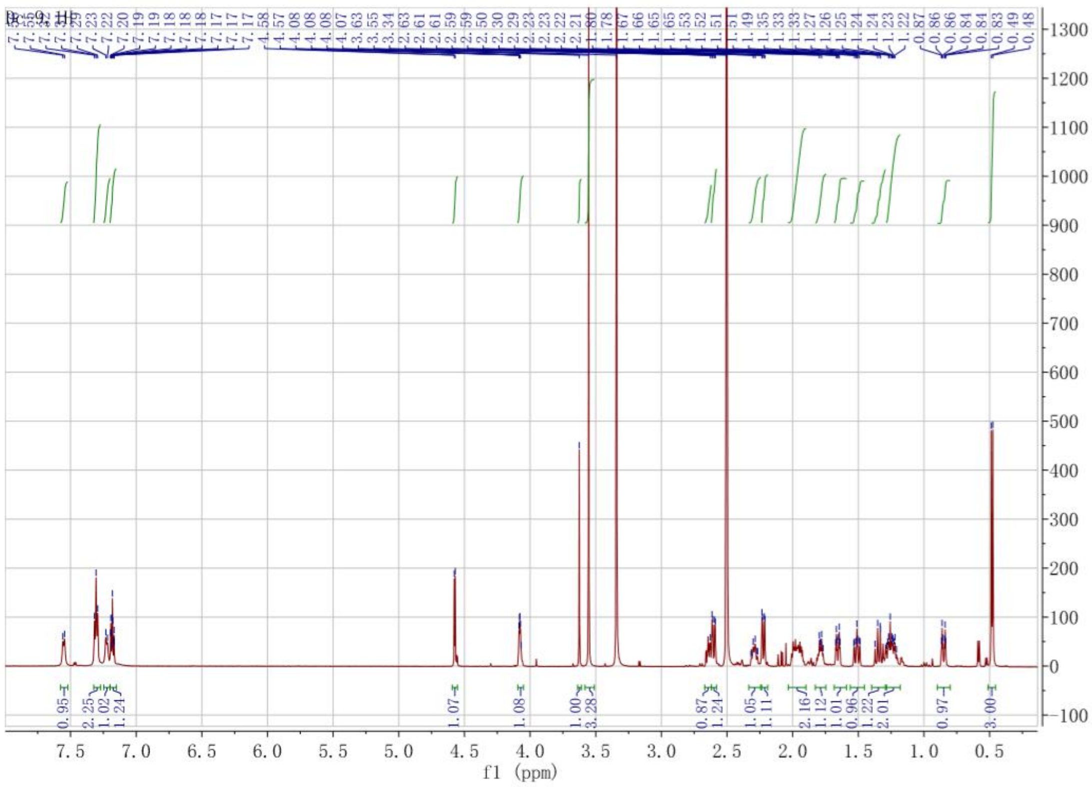

**Fig S21.** $^{1}$HNMR spectrum of (**3**) in DMSO-$d_6$

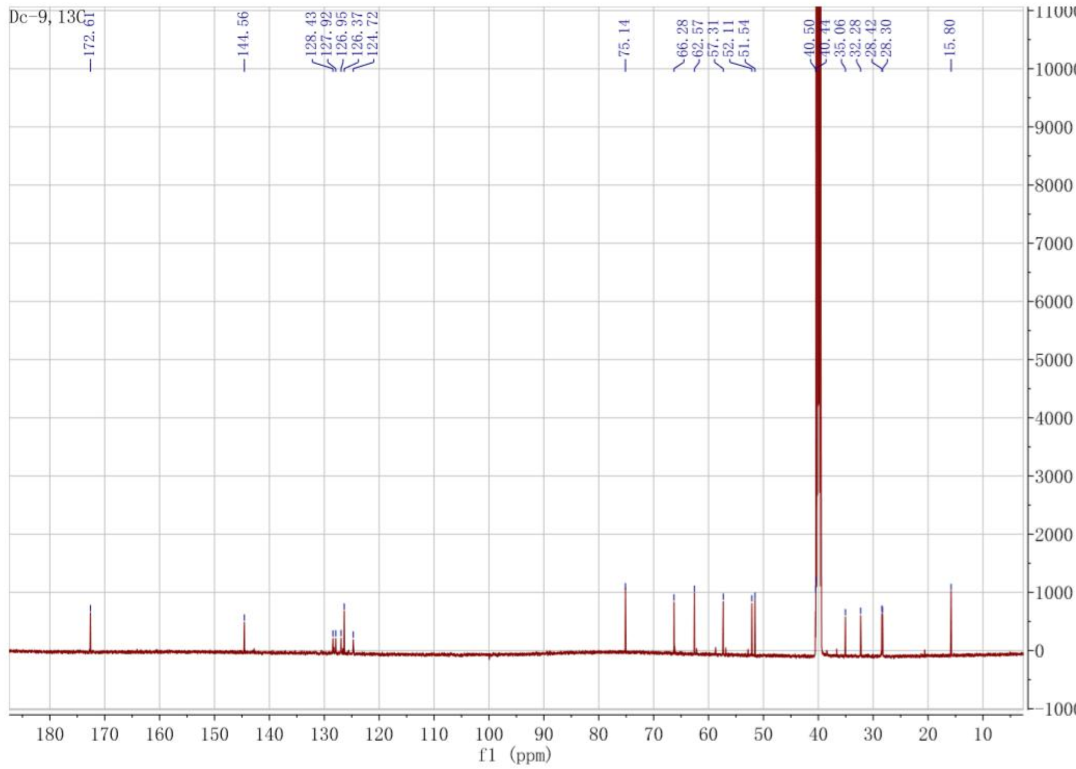

**Fig S22.** $^{13}$C NMR spectrum of (**3**) in DMSO-$d_6$

Table S1 NMR Spectroscopic Data of **3** in (DMSO-$d_6$) ($\delta$ in ppm and J in Hz) [a]

| Pos | **3** | |
|---|---|---|
| 1 | 28.4, $CH_2$ | 1.35, m<br>1.25, m |
| 2 | 28.3, $CH_2$ | 1.95, m |
| 3 | 62.6, CH | 2.28, m |
| 5 | 52.1, CH | 2.60, dd (3.3, 11.4) |
| 6 | 32.3, $CH_2$ | 1.51, ddd (3.0, 11.4, 14.4)<br>1.30, m |
| 7 | 66.3, CH | 4.08, m |
| 8 | 51.5, CH | 2.22, dd (2.4, 10.8) |
| 9 | 57.3, CH | 2.65, m |
| 10 | 172.6, qC | |
| 11 | 40.4, $CH_3$ | 3.55, s |
| 12 | 75.1, qC | |
| 13 | 40.5, CH | 1.78, m |
| 14 | 35.1, $CH_2$ | 1.66, dt (3.6, 3.6, 12.6) |
| | | 0.85, dt (3.0, 3.0,13.8) |
| 15 | 15.8, $CH_3$ | 0.48, d (6.6) |
| 1' | 144.6, qC | |
| 2'/6' | 124.7, CH | 7.55, br d (7.8) |
| 3'/5' | 127.0, CH | 7.31, t (7.2, 7.2) |
| 4' | 128.4, CH | 7.22, br d (7.8) |
| 6-OH | | |
| 7-OH | | 4.57, d (4.2) |
| 12-OH | | 3.63, s |

[a] NMR spectroscopic data were recorded at 600 MHz ($^1$H NMR), 150 MHz ($^{13}$C NMR).
